# Peritoneal Modulators of Endometriosis-Associated Ovarian Cancer

**DOI:** 10.3389/fonc.2021.793297

**Published:** 2021-11-25

**Authors:** Sarah Brunty, Lauren Clower, Brenda Mitchell, Taylor Fleshman, Nadim Bou Zgheib, Nalini Santanam

**Affiliations:** ^1^ Department of Biomedical Sciences, Joan C. Edwards School of Medicine, Marshall University, Huntington, WV, United States; ^2^ Department of Obstetrics & Gynecology, Joan C. Edwards School of Medicine, Marshall University, Huntington, WV, United States

**Keywords:** peritoneal fluid, FoxP3, EZH2, endometriosis, ovarian cancer

## Abstract

Ovarian cancer is the 4th largest cause of cancer death in women. Approximately 10–15% of women of childbearing age suffer from endometriosis. Endometriosis is defined by the growth and presence of endometrial tissue (lesions) outside of the uterus. The women with endometriosis also have an increased presence of peritoneal fluid (PF) that comprises of inflammatory cells, growth factors, cytokines/chemokines, etc. Epidemiological studies have shown that >3% of women with endometriosis develop ovarian cancer (low-grade serous or endometrioid types). Our hypothesis is that the PF from women with endometriosis induces transformative changes in the ovarian cells, leading to ovarian cancer development. PF from women with and without endometriosis was collected after IRB approval and patient consent. IOSE (human normal ovarian epithelial cells) and TOV-21G cells (human ovarian clear cell carcinoma cell line) were treated with various volumes of PF (no endometriosis or endometriosis) for 48 or 96 h and proliferation measured. Expression levels of epigenetic regulators and FoxP3, an inflammatory tumor suppressor, were determined. A Human Cancer Inflammation and Immunity Crosstalk RT^2^ Profiler PCR array was used to measure changes in cancer related genes in treated cells. Results showed increased growth of TOV-21G cells treated with PF from women with endometriosis *versus* without endometriosis and compared to IOSE cells. Endo PF treatment induced EZH2, H3K27me3, and FoxP3. The RT^2^ PCR array of TOV-21G cells treated with endo PF showed upregulation of various inflammatory genes (TLRs, Myd88, etc.). These studies indicate that PF from women with endometriosis can both proliferate and transform ovarian cells and hence this microenvironment plays a major mechanistic role in the progression of endometriosis to ovarian cancer.

## Introduction

Epithelial ovarian cancer (EOC), which is the leading cause of death in women with gynecological malignancies, is very difficult to diagnose and treat due to its asymptomatic presentation and insufficient knowledge on factors that initiate tumorigenesis (1–3). It comprises over 95% of existing ovarian cancers (4) and women have a one in 78 chance of developing EOC (5). Women with EOC have poor prognosis with 61% of cases being detected at advanced stages with a 5-year survival rate of only 27% (1, 2). EOC includes a high-grade serous, low-grade serous, mucinous, an endometrioid, and a clear-cell subtype (6). The endometrioid or clear cell carcinoma represents the main types of endometriosis-associated ovarian cancer (EAOC) that may develop from precursor endometriotic lesions in the ovary. EAOC has an odds ratio of 1.42 for the progression to ovarian cancer in the presence of endometriosis (7, 8). This odds ratio greater than one clearly demonstrates that exposure to endometriosis is a major risk factor for developing EOC. However, the reason for this increased risk of developing ovarian cancer from the progression of endometriosis currently remains unclear.

Endometriosis is an estrogen-dependent chronic inflammatory gynecological disorder that often leads to debilitating symptoms including chronic pelvic pain, menstrual irregularities, dysmenorrhea, and dyspareunia. Approximately 10% of women of childbearing age suffer from endometriosis (9). Similar to EOC, 20–25% of patients remain asymptomatic which contributes to a delay in diagnosis and treatment (10). There are various theories for the etiology and pathogenesis of endometriosis with the most widely accepted hypothesis being Sampson’s retrograde menstruation which is characterized by the development of ectopic endometrial cell implants in the peritoneum (11, 12). Endometrial cells can also escape from immune clearance, attach and invade the peritoneal epithelium, and play a role in angiogenesis (13). While endometriosis is often considered a benign condition, endometrial implants exhibit several molecular and histopathological characteristics similar to those demonstrated by neoplastic cells (14). These characteristics include metastasis to distant sites, invasion and migration, and the establishment of neurovascularity. In addition to this pathogenesis, researchers have found increased levels of inflammatory peritoneal fluid in women suffering from endometriosis (13) which we propose is a main contributor for malignant transformation to EOC.

The peritoneal fluid of women with endometriosis is characterized by increased levels of cytokines, chemokines, growth factors, pain-inducing molecules, and inflammatory cells. Increased amounts of prostaglandins, activated macrophages, IL-1, IL-6, IL-10, and tumor necrosis factor alpha (TNF-α) have been found in the PF of patients with endometriosis when compared to the PF of control groups (15–17). Other studies have shown that the increased levels of PF contribute to sustained peritoneal inflammation which regulates both the growth and proliferation of endometrial lesions (18). In patients with endometriosis, the NK cells present in the PF has a defect in its cytotoxic function, hence these cells are unable to remove the endometriotic implants leading to endometriosis. There is also a proposed lack of tissue clearance due to an increase in immunosuppressive regulatory T cells (T_regs_) present in the endometrial PF (18). These increased levels of FoxP3+ Tregs decrease immune recognition and clearance of endometrial antigens leading to increased endometrial implants at ectopic sites (19, 20). The escape of endometrial antigens from immune surveillance and increased implantation is important as some of these endometriotic implants may navigate towards the ovaries where they can undergo malignant transformation under the environment of the endometriotic milieu or remain in the peritoneal environment and undergo transformation. In ovarian cancer, increased expression of FoxP3 has been shown to correlate with reduced survival time and disease progression (21). However, some other studies have shown that when FoxP3 is upregulated, this inhibited disease progression for EOC (22). Hence, a better understanding of this pathway in EOAC is essential.

Dissemination of EOC follows a non-traditional invasion–migration cascade by forming loosely attached outgrowths that transit through the peritoneal fluid and attach to new sites. Studies have suggested that the metastasis of ovarian carcinoma is less complicated compared to other types of malignancies as the cancer cells break off individually or in clusters from the original site and passively disseminate to other locations in the peritoneum (23). The ovarian cancer cells invade the mesothelium covering all structures in the peritoneum as its primary microenvironment (23). This invasion migration cascade is very similar to the one seen in endometriosis representing common features present in both conditions.

Endometriosis and EOC may have some relation since they both pertain to pelvic tissues that grow uncontrollably. We propose that this connection between endometriosis and its malignant progression to EOC may be the result of these PF microenvironmental changes which induce cancer-related genetic alterations. Recently, our laboratory has shown that in endometriosis, the PF regulates epigenetic pathways to induce the growth of these lesions (24). We showed that when PF from women with endometriosis was added to endometrial cells, there was an increase in the PRC2 complex, specifically EZH2, and its target H3K27me3. Additionally, there was an increase in miR-155/FoxP3 expression which may be working in coordination with the PRC2 complex to drive the progression of endometriosis. While we believe that these mechanisms may also be behind the transformation of endometriosis to EOC, there is insufficient knowledge that the PF milieu in EOC has similar properties as the PF milieu in endometriosis. Based on these findings, we hypothesize that the endometriotic PF microenvironment would induce inflammatory and epigenetic pathways resulting in transformative changes in the ovarian or endometrial cells leading to the development of EAOC. In our present study, we used a human ovarian clear cell carcinoma line, TOV-21G, treated with PF from women with endometriosis compared to women without endometriosis to discover the effects of the endometrial PF milieu on ovarian cell growth and proliferation.

## Materials and Methods

### Human Subject Participants

Women ages 21 to 60 years, undergoing tubal ligation or having non-endometriosis disorders (controls) or patients with endometriosis (“endo”, laparoscopically diagnosed followed by pathological confirmation and/or patients with symptoms) were recruited from the Obstetrics-Gynecology clinic at the Cabell Huntington Hospital, Joan C Edwards School of Medicine, Marshall University, in Huntington, WV. In this study, endo patients were diagnosed with stage I/II and pathologically confirmed to exhibit peritoneal endometriosis. This HIPAA compliant study was approved by the Institutional Review Board of the Marshall University School of Medicine and was carried out per the principles of the Declaration of Helsinki. All patients were consented prior to the study. All women completed a gynecologic/infertility history form, a pre-operative quality of life questionnaire and assessment of pain using a visual analog scale for assessment of endometriosis associated pain (dysmenorrhea, non-menstrual pelvic pain, dyspareunia, and dyschesia) (adapted from the validated International Pelvic Pain Society’s Pelvic Assessment Form). Date of their last menstrual period was used to assess their cycle time. The inclusion criteria included women ages 21–60 years old, with normal menstrual cycles and otherwise in normal health (except for pain and endometriosis) who have not been on any hormonal medication for at least one month before sample collection. Exclusion criteria included subjects with current medical illnesses such as diabetes, cardiovascular disease, hyperlipedemia, hypertension, systemic lupus erythematosis or rheumatologic disease, positive HIV/AIDS, active infection. Subjects were asked to stop multivitamins that contain high levels of antioxidants and anti-inflammatory medications one month prior to sample collection. Peritoneal fluid (PF) from both women with and without endometriosis were collected during the surgery through the aspiration from the peritoneal/abdominal cavity and without the use of saline. Prior to use, PF (devoid of blood contamination) was spun at 2000 x g to remove any cellular debris. The supernatant, cell-free PF was used immediately for studies or stored at -80 C for future use.

### Cell Culture

TOV-21G, human ovarian clear cell carcinoma cell line (CRL-11730, ATCC, Manassas, MA), and IOSE364, human normal ovarian epithelial cells (25, 26) (gift from Dr. Charlie Chen, Aldous-Broadus College), were cultured in T75 flasks in complete media (MCDB 105, Medium 199 (1:1), 15% FBS, 1% Pen/Strep, 1% glutamine). When cells were approximately 80% confluent, media was changed to 1.5% charcoal-stripped FBS containing media before being treated with 1% or 10% peritoneal fluid from women with and without endometriosis (endo and control PF respectively) for 48 h.

### xCELLigence Cell Proliferation Studies

TOV-21G cells were used to test cell proliferation under various conditions using xCELLigence technology (Cat No: 05469759001, Agilent, Santa Clara, CA). This technology uses modified 16-well plates (E-plates, Cat No: 5469813001, Agilent, Santa Clara, CA) in which microelectrodes are attached at the bottom of the wells in which cell impedance or cell index (CI) can be measured. For cell proliferation studies, 10,000 cells per well were plated in 100 μl of complete media in E-Plates and placed on the xCELLigence reader for 24 h. After 24 h, media was removed in all wells and 1.5% charcoal-filtered FBS containing media added before treating with 1 or 10% of PF (endometriosis or control) and followed in the xCELLigence reader for another 96 h. Readings were taken once every hour throughout the whole experiment for 96 h. All treatments were performed in triplicate and change in CI averages with PF treatments were compared to media only treated cells and represented as CI averages or percentage of growth. The more number of cells present in the wells (due to proliferation or growth) the higher the CI measurement.

### Expression of EZH2 and H3K27me3

IOSE364 cells and TOV-21G cells treated with peritoneal fluid alone (1 or 10%) for 48 h were suspended in TRI reagent before extraction of the RNA from the cells. The quantity and quality of the mRNA, was measured using the NanoDrop 2000 spectrophotometer. cDNA synthesis of 1 μg of mRNA was done using the iScript cDNA synthesis Kit (1708890-Biorad, Hercules, CA). mRNA expression was analyzed from the freshly created cDNA samples using SYBR Green (1725270-Biorad, Hercules, CA) for EZH2 (F:AAGGAGTTTGCTGCTGCTCT;R: ATTAATGGTGGGGGTGCTGG) and 18S as housekeeping gene (F: GCAATTATTCCCCATGAACG; R: GGCCTCACTAAACCATCCAA). H3k27me3 was determined using the EpiQuik Histone modification multiplex assay (Epigentek, P-3100, Farmingdale, NY).

### RT^2^ Profiler PCR Cancer Array

TOV-21G cells that have been treated with 1 or 10% control or endometrial PF were used to measure the fold-changes of human cancer genes present in the Human Cancer Inflammation and Immunity Crosstalk RT^2^ Profiler PCR Array (PAHS-181Z, Qiagen). IOSE364 (normal ovarian epithelial cells) were also treated with control or endometriotic PF and ran for comparison.

## Results

### Proliferation and Migration of Ovarian Cancer Cells Using Cell Impedance Method

**Figure 1:**
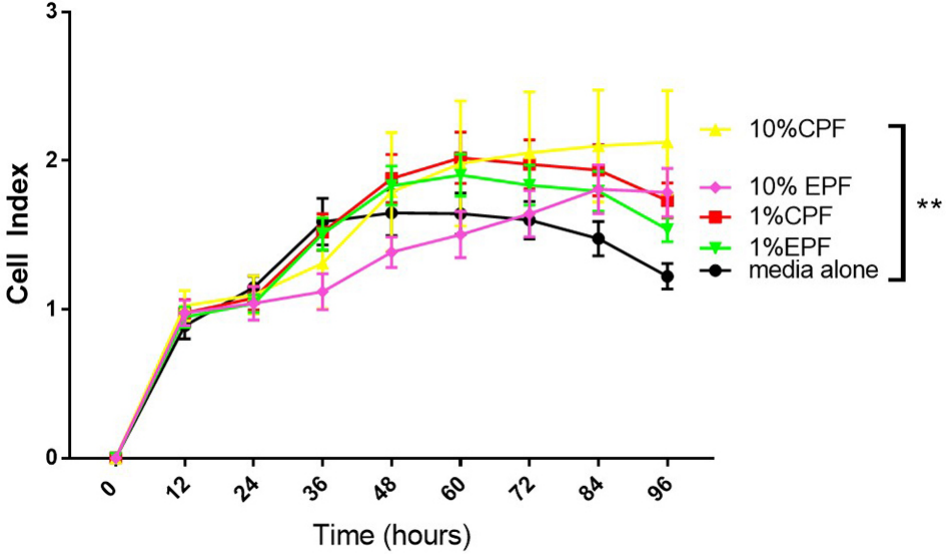
Peritoneal fluid increases proliferation of TOV-21G cells as determined by increase in Cell Index. **p < 005.

In this experiment, we tested the ability of PF (from patients with-EPF or without endometriosis-CPF) to increase the proliferation of ovarian cells (TOV-21G) using the xCELLigence technology (RTCA DP). This is a technology based on cell impedance changes that allows for more accurate, real-time measurements of cell growth without using a reagent such as Reliablue (27). This system uses specific plates that have gold microelectrodes attached to the bottom that produce electrons, which interact with the solution as they complete their circuit. As the number of adhered cells in the plate increases, they interfere with the flow of electrons. This allows for the proliferation of the cells to be measured over time. The impedance read out is measured as cell index (CI). **Figure 1** shows that human ovarian clear cell carcinoma cells (TOV-21G) cells when exposed to 1 or 10% PF (CPF or EPF = n = 6–8) had increased proliferation with increasing concentrations. The significance (one-way ANOVA) was reached with 10% PF concentration (p = 0.0012). These observations suggest that PF could increase proliferation of TOV-21G ovarian clear-cell cancer cells.

### PF Mediated Induction of EZH2-H3k27me3 in Ovarian Cells

**Figure 2:**
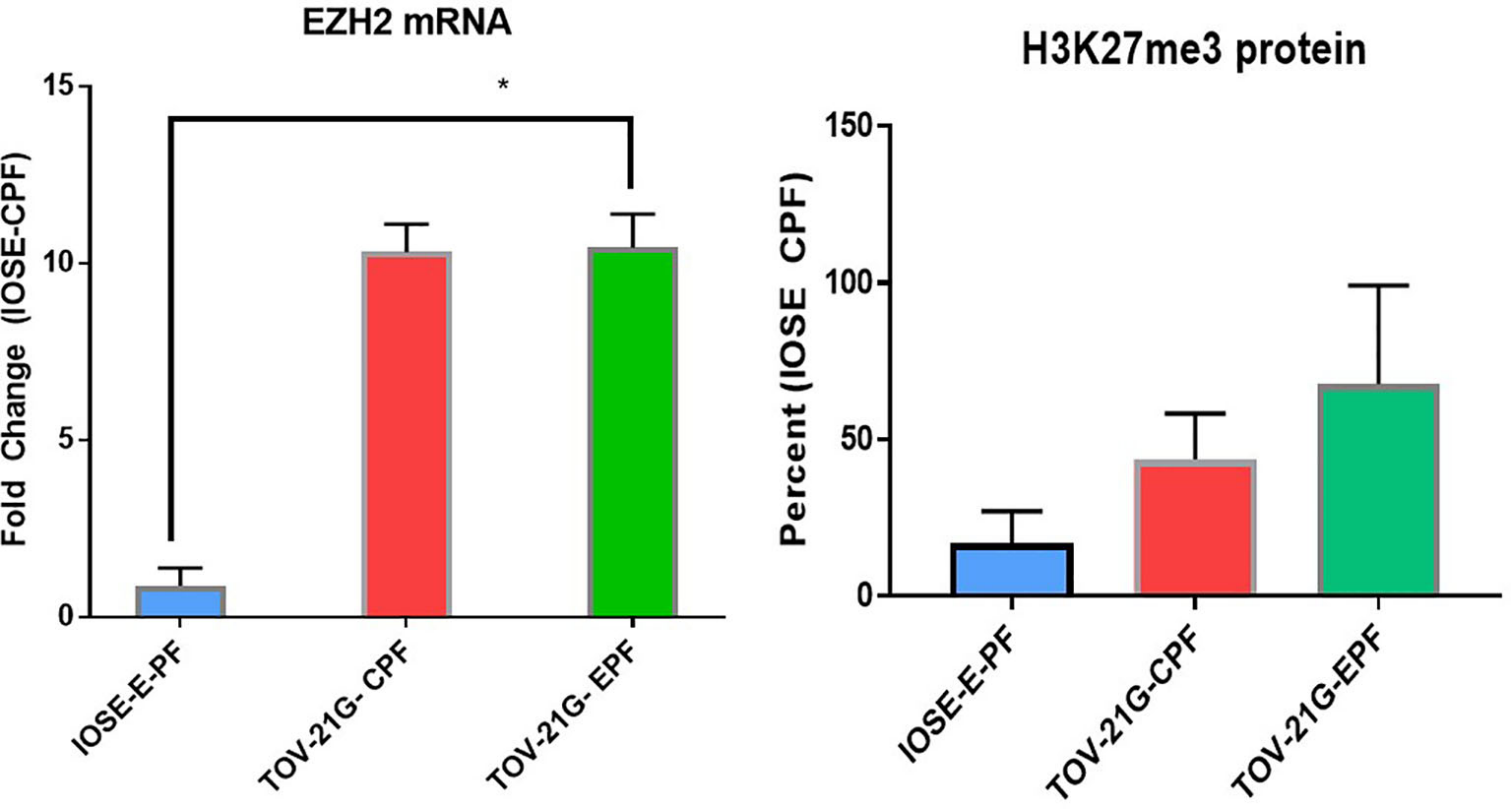
Peritoneal fluid modulates the expression of EZH2/H3k27me3 in IOSE and TOV-21G cells. *p < 0.05.

We recently showed the PF can modulate the EZH2/H3k27me3 axis in endometriosis (24). **Figure 2** shows that 1% Ctrl or endo PF (n = 6–8) at 48 h, also induced EZH2 and H2K27me3 in IOSE and TOV-21G cells (**Figure 2**). EZH2 was measured using real time PCR and H3K27me3 protein was measured using ELISA kit (Epigentek). One-way ANOVA showed an increase in both EZH2 (p = 0.0341) and H3k27me3 expression in endo PF treated TOV-21G cells.

### Increase in FoxP3 and TLR Pathway Gene Expression in PF Treated Ovarian Cells

**Figure 3:**
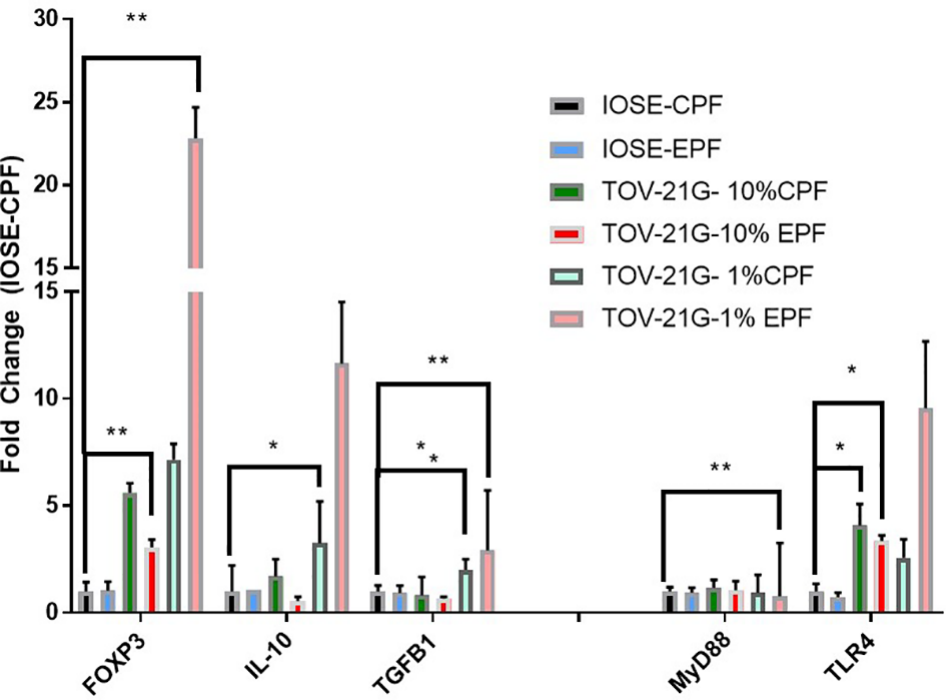
Peritoneal fluid induces FoxP3 and TLR pathway genes in TOV-21G cells. *p < 05; **p < 0.005.

IOSE364 cells (immortalized normal ovarian epithelial cells) and TOV-21G (clear cell ovarian carcinoma cell line) exposed to 1% PF from women with or without endometriosis (n = 6–8) at 48 h showed increased expression of FoxP3, which was induced significantly (t-test) by EPF (p = 0.016) and a trend in increased expression of Treg markers IL-10 and TGFb. There was also induction in FoxP3 downstream genes that are related to the TLR pathway (MyD88) (p = 0.02) and TLR4 (p = 0.0009) in endo PF treated TOV-21G cells compared to IOSE-CPF treated cells (**Figure 3**).

RT^2^ Profiler PCR array for cancer related genes also showed upregulation of several other inflammatory genes including IL-6, CCL4, MCP-1, etc. (**Table 1**). The induction was more prominent in 1% endo PF treated cells compared to 10% PF treated cells and higher in TOV-21G treated cells compared to IOSE cells.

**Table 1:** Significantly regulated cancer related genes in PF treated IOSE or TOV-21G cells.

Gene Symbol	IOSE-1% EPF		TOV-21G 10% CPF		TOV-21G 10% EPF		TOV-21G 1% CPF		TOV-21G 1% EPF	
	Fold Change	P-Value	Fold Change	P-Value	Fold Change	P-Value	Fold Change	P-Value	Fold Change	P-Value
**ACKR3**	0.9	0.5514	0.46	0.1394	0.16	0.0373	0.6	0.4220	**2.95**	0.3059
**AICDA**	1.08	0.8386	**9.4**	0.0130	**5.37**	0.0153	**14.45**	0.1121	**34.69**	0.0523
**BCL2**	0.73	0.4132	**11.65**	0.0190	**7.86**	0.0009	**15.35**	0.0441	**35.26**	0.0040
**BCL2L1**	0.99	0.8316	1.05	0.7236	0.85	0.2316	1.73	0.0770	**2.44**	0.0038
**CCL2**	0.98	0.7723	0.15	0.0307	0.22	0.0378	0.21	0.0363	0.15	0.0127
**CCL20**	0.92	0.6738	**12.46**	0.0002	**45.4**	0.0020	**14.39**	0.1013	**11.64**	0.0129
**CCL21**	1.86	0.7287	**80.97**	0.0857	**46.68**	0.0392	**275.64**	0.1043	**429.5**	0.0145
**CCL28**	0.98	0.7052	**13.79**	0.0039	**12.54**	0.0073	**9.83**	0.0020	**9.46**	0.0007
**CCL5**	0.85	0.5720	0.12	0.0020	0.06	0.0017	0.34	0.0437	0.39	0.7172
**CCR1**	1.3	0.6283	**44.51**	0.0918	**25.66**	0.0004	**97.91**	0.0829	**460.59**	0.0129
**CCR10**	1.14	0.6051	1.36	0.3900	0.79	0.2576	**3.56**	0.0237	**9.87**	0.0002
**CCR4**	1.47	0.6913	**3**	0.2439	0.95	0.9226	**3.37**	0.0267	**14.19**	0.1749
**CCR7**	0.78	0.4439	**3.48**	0.1537	**2.7**	0.0825	**17.59**	0.0293	**39.57**	0.0470
**CCR9**	0.58	0.3730	**2.87**	0.2317	0.78	0.5246	**3.66**	0.1344	**7.34**	0.0358
**CD274**	0.98	0.9149	0.28	0.0338	0.26	0.0294	0.42	0.0585	0.49	0.2404
**CSF1**	0.98	0.7800	0.31	0.0094	0.34	0.0094	1.29	0.3403	1.26	0.4552
**CSF2**	0.87	0.4291	0.02	0.0033	0.02	0.0032	0.19	0.0088	0.19	0.0068
**CSF3**	0.87	0.5140	0blue	0.0028	0	0.0028	0	0.0029	0.01	0.0006
**CXCL1**	1.13	0.5694	0.53	0.0373	1.06	0.7493	0.31	0.0044	0.24	0.0008
**CXCL12**	0.74	0.1908	0.44	0.0348	0.27	0.0143	1	0.6482	2.08	0.1982
**CXCL2**	1.07	0.8069	1.1	0.7145	1.79	0.0251	0.46	0.0285	0.4	0.0425
**CXCL5**	1.15	0.5493	**45.65**	0.0035	**113.35**	0.0007	**28.84**	0.0018	**25.93**	0.0001
**CXCL9**	1.77	0.3672	**3.35**	0.0919	1.7	0.2576	**2.57**	0.2206	**9.87**	0.0138
**CXCR1**	1.08	0.8386	1.87	0.4346	0.64	0.4363	**3.92**	0.0361	**14.09**	0.2290
**CXCR2**	1.31	0.2199	**11.15**	0.0906	**6.86**	0.0012	**17.27**	0.0926	**51.83**	0.0020
**CXCR3**	0.69	0.4542	**7.32**	0.1016	**5.27**	0.0006	**6.63**	0.1294	**28.62**	0.0236
**CXCR4**	0.94	0.8880	**382.5**	0.0002	**590.04**	0.0044	**434.54**	0.0229	**413.91**	0.0022
**CXCR5**	0.69	0.3086	**11.6**	0.1105	**5.66**	0.0141	**15.82**	0.1855	**67.56**	0.0161
**EGF**	1.18	0.8818	**5.23**	0.0320	**3.69**	0.0032	**3.56**	0.1872	**14**	0.0047
**EGFR**	0.73	0.2249	**4.53**	0.0010	**5.07**	0.0005	**6.68**	0.0033	**7.83**	0.0011
**FASLG**	1.4	0.6884	**5.75**	0.1175	**2.49**	0.1228	**4.91**	0.0928	**23.82**	0.0416
**GBP1**	1.12	0.5361	0.24	0.0012	0.24	0.0013	0.41	0.0182	0.46	0.0201
**GZMB**	0.5	0.4130	**44.2**	0.1183	**20.99**	0.0084	**83.09**	0.1488	**241.46**	0.0019
**HIF1A**	0.63	0.1302	**28.89**	0.0005	**27.06**	0.0008	**21.41**	0.0000	**19.88**	0.0001
**HLA-A**	1.24	0.2260	0.3	0.0111	0.27	0.0073	0.81	0.3066	0.93	0.7452
**HLA-B**	1.02	0.8507	**148.33**	0.0071	**154.13**	0.0000	**440.6**	0.0043	**652.88**	0.0046
**HLA-C**	1.13	0.7111	**22.2**	0.1304	**17.61**	0.0001	**24.31**	0.0938	**94.62**	0.0146
**IGF1**	0.67	0.2399	**5.07**	0.0801	1.97	0.0771	**4.67**	0.1776	**22.97**	0.0357
**IL12A**	1.13	0.4419	**4.3**	0.0093	**8.14**	0.0104	**8.21**	0.0170	**9.18**	0.0000
**IL12B**	5.7	0.3744	**4.05**	0.2411	0.85	0.8802	**3.24**	0.0143	**16.7**	0.2296
**IL13**	0.37	0.3177	**2.21**	0.5989	1.26	0.6958	**2.38**	0.4545	**11.18**	0.0416
**IL15**	1.05	0.7589	**2.47**	0.0121	**2.01**	0.0106	**6.53**	0.0000	**5.81**	0.0009
**IL17A**	1.08	0.8386	**2.09**	0.4074	1.08	0.9414	**3.92**	0.0361	**14.71**	0.2553
**IL1A**	1.18	0.4815	0.11	0.0070	0.08	0.0062	0.1	0.0068	0.09	0.0019
**IL1B**	1.24	0.3081	0.01	0.0007	0	0.0007	0.01	0.0007	0.01	0.0001
**IL2**	1.08	0.8386	**5.62**	0.3032	**2.75**	0.0953	**3.38**	0.0264	**18.85**	0.1903
**IL23A**	1.25	0.3381	0.32	0.0218	0.21	0.0118	0.92	0.6615	0.99	0.9166
**IL6**	1.32	0.1886	1.43	0.2205	**2.28**	0.0009	0.86	0.3864	0.57	0.0448
**CXCL8**	1.02	0.9683	0.03	0.0035	0.05	0.0038	0.05	0.0038	0.07	0.0009
**IRF1**	1.01	0.8830	0.1	0.0001	0.07	0.0001	0.34	0.0007	0.44	0.0080
**KITLG**	1.02	0.9140	**78.21**	0.0041	**107.49**	0.0010	**41.55**	0.0001	**38.74**	0.0013
**MICA**	1.16	0.4079	**9.6**	0.0023	**10.44**	0.0000	**11.31**	0.0028	**8.86**	0.0002
**MICB**	1	0.9877	1.99	0.0068	**2.08**	0.0005	**2.71**	0.0004	**2.49**	0.0078
**MIF**	0.92	0.6228	**2.16**	0.0259	1.66	0.0377	**2.12**	0.0083	1.72	0.0475
**MYC**	0.97	0.8982	**3.17**	0.0001	**2.75**	0.0005	**2.43**	0.0137	**3.02**	0.0070
**NFKB1**	1.14	0.3628	0.58	0.0033	0.55	0.0039	0.72	0.0008	1.01	0.8868
**NOS2**	1.3	0.8448	**30.82**	0.0811	**18.44**	0.0094	**60.83**	0.1342	**184.8**	0.0048
**PDCD1**	0.93	0.9686	**4.6**	0.0028	**3.98**	0.0005	**7.78**	0.0792	**19**	0.0338
**PTGS2**	0.81	0.3824	0.04	0.0032	0.01	0.0008	0.01	0.0008	0.02	0.0001
**STAT1**	1.05	0.6698	**4.67**	0.0000	**5.01**	0.0000	**3.47**	0.0006	**4.07**	0.0010
**STAT3**	1.18	0.3313	1.76	0.0031	1.77	0.0053	**3.25**	0.0001	**3.4**	0.0002
**TLR3**	1.04	0.8182	**5.39**	0.0014	**6.21**	0.0000	**3.15**	**0.0029**	**3.55**	0.0289
**TNF**	1.51	0.4840	**1112.3**	0.0001	**992.32**	0.0000	**1606.8**	0.0000	**1639.5**	0.0009
**TNFSF10**	0.86	0.4543	0.33	0.0719	0.23	0.0150	0.28	0.0224	0.91	0.7145
**TP53**	1.01	0.9680	0.28	0.0011	0.28	0.0012	0.28	0.0011	0.34	0.0005

Bold numbers in the table represents upregulation of the gene expression. Blue font represents downregulation of gene expression. Red font represents significant p value <0.05.

## Discussion

Through these studies presented here, we provided evidence that endometriotic PF increases proliferation, induces inflammatory genes (including FoxP3 mediated signaling) and modulates EZH2/H3K27me3 in human clear-cell ovarian carcinoma cell lines (TOV-21G) compared to normal ovarian epithelial cells (IOSE). This provides insights into the possible mechanisms by which endometriotic peritoneal milieu by modulating epigenetic pathways may promote endometriosis associated cancers.

Inflammation is a key player in both endometriosis (28–30) and ovarian cancer (31–33). Patients with endometriosis, have an impaired endometrium and an inflamed peritoneal microenvironment. In endometriosis, peritoneal fluid (PF) is a highly dynamic microenvironment that is in a constant state of inflammation (34–37). There is a similar increase in PF accumulation in patients with ovarian cancer. Cytologic examinations of peritoneal washings are a common prognostic (as well as epigenetic) tool in ovarian cancer (38, 39). An increased presence of immune cells including monocyte-macrophages, and T_regs_ is quite common in the peritoneal microenvironment (40–43). We have earlier shown increased presence of inflammatory and redox markers in the PF of women with endometriosis compared to control women (44–50). We also showed that endometrial cells treated with PF from women with endometriosis induced colony stimulatory factor-1 (44) and monocyte chemotactic protein-1 (47).

Forkhead box protein P3 (FoxP3 T_regs_) cells are essential for maintenance of immune tolerance (51, 52). However, the expression and function of FoxP3 in cancer cells is contradictory, since in pancreatic cancer and melanoma it is expressed in the tumor cells whereas it is present in the normal epithelial cells in human breast and prostate cancer (53–56). Furthermore, FoxP3 can act both as a tumor suppressor as seen in breast cancer (57) but also enables cancer cells to prevent T-cell responses directed towards them and hence results in tumor progression and poor prognosis (58, 59). A higher percentage of FoxP3 T_regs_, and immunosuppressive cytokines, interleukin-10 (IL-10), transforming growth factor beta (TGF-β) levels are present in the PF and increased expression in endometriotic tissue of patients with endometriosis compared to control women (43, 60). In endometriosis, increased FoxP3 T_reg_ response is suggested to lead to cancer progression (61). A recent study showed that endometriotic PF increased the recruitment of FoxP3-Tregs in the peritoneal microenvironment (19) We observed an induction of FoxP3 levels in ovarian cells treated with endometriotic PF, thus, it is plausible that though it is an important component of T_reg_ cells and essential in immune homeostasis, it may also enable cancer cells to escape T cell responses thus resulting in tumor progression and poor prognosis. There are suggestions in the literature that in endometriosis, increased FoxP3 T_reg_ response may lead to cancer progression (19, 61, 62).

Our interest in FoxP3 is also related to its ability to associate with the polycomb proteins EZH2/PRC2 complex and play a role in epigenetic regulation (63–67) and modulating the expression of tumor associated genes The polycomb group of proteins (PRC1 and PRC2) plays important roles in cell growth and proliferation (68, 69). EZH2/PRC2 complex catalyzes trimethylation of lysine 27 of histone H3 (H3K27me3) which then interacts with the chromatin complex resulting in gene repression (70–72). EZH2 is overexpressed in patients with EOC (68, 73–76) and promotes proliferation, inhibits apoptosis, and enhances angiogenesis in ovarian cancer (77). Inhibition of EZH2, inhibits the growth of ovarian cancer (77–80). In most cancers, there is a reduction in FoxP3 and increased EZH2, however, we recently showed that endometriotic PF promoted a cross-talk between EZH2 and FoxP3 (24). We found that endo PF increased the expression of EZH2/H3K27me3 in TOV-21G cells, suggesting a plausible interaction with the inflammatory modulators such as FoxP3.

**Figure 4:**
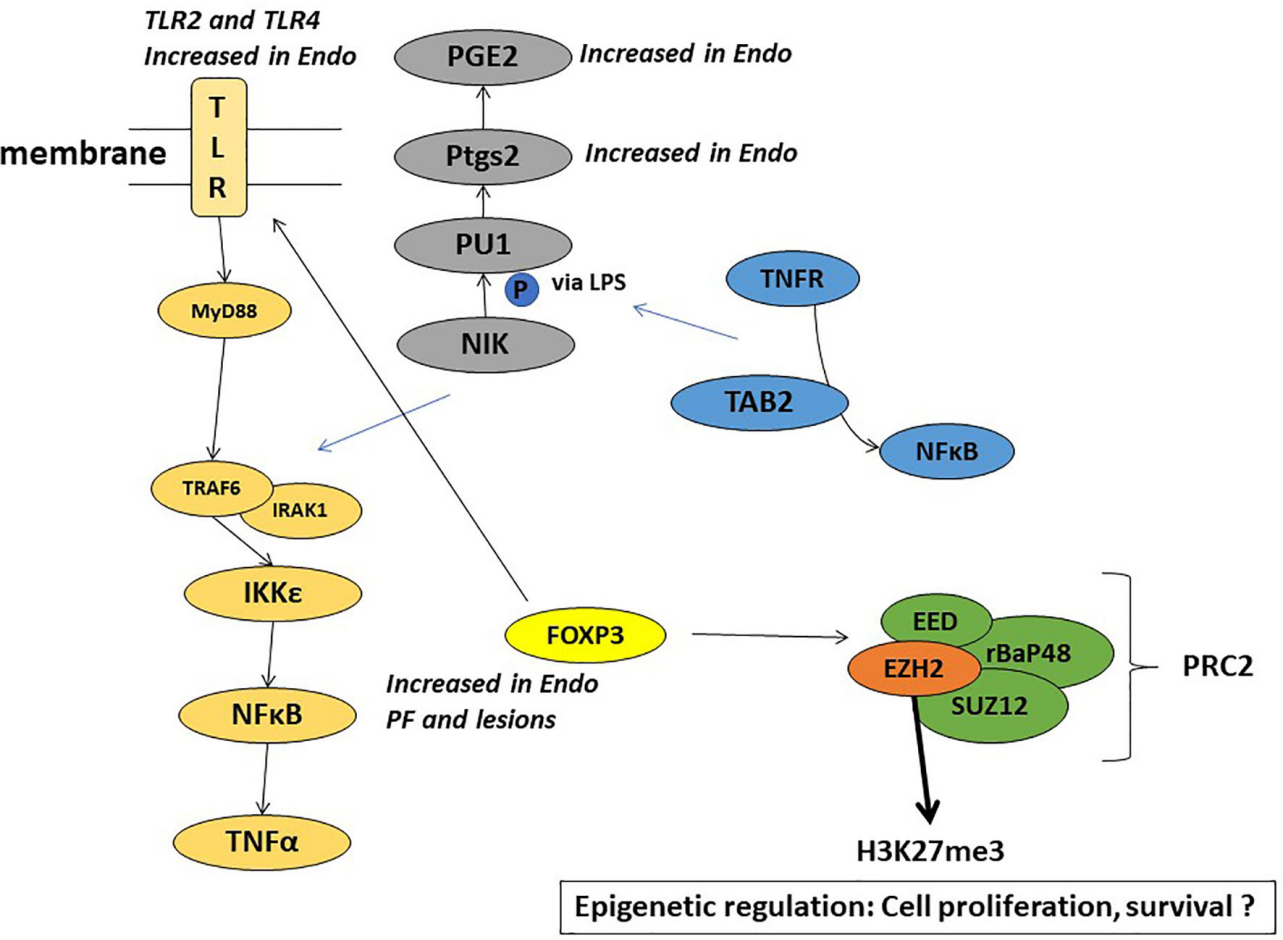
Schematic representation of the plausible mechanisms involved in the role of endometriotic PF in EAOC.

Toll like receptors (TLR2 and TLR4), signal through the adaptor molecule, myeloid differentiation primary response gene 88 (MyD88) leading to nuclear translocation of NF-kB and upregulation of pro-inflammatory genes such as IL-6, CCL4 (Treg released cytokine) and CCL2 (MCP-1). The TLR pathway interacts with FoxP3-Treg in immune response (81, 82) and plays a role in chemoresistance (83, 84). Our study showed endometriotic PF induced several of these inflammatory genes involved in cancer pathways, including the TLR pathway (63, 64, 67). **Figure 4** highlights some of the mechanistic pathways (TLR pathway, NFkB activation, and Cox-2 activated prostaglandin mediated proliferation) that are possible targets of FoxP3-EZH2 crosstalk and that may be at play in endometriosis associated ovarian cancer. More studies are needed to understand the importance of these pathways in the etiology of EAOC, in order to develop early diagnostic or treatment options for this condition.

## Data Availability Statement

The original contributions presented in the study are included in the article/supplementary material. Further inquiries can be directed to the corresponding author.

## Ethics Statement

The studies involving human participants were reviewed and approved by the Marshall University IRB. The patients/participants provided their written informed consent to participate in this study.

## Author Contributions

Conceptualization, NS. Data curation, SB and NS. Formal analysis, SB and NS. Funding acquisition, NS. Investigation, SB and NS. Methodology, SB, LC and NS. Project administration, NS. Resources, BM, NZ and NS. Supervision, NS. Validation, SB, TF and NS. Writing—original draft, SB, LC and NS. Writing—review and editing, NS. All authors contributed to the article and approved the submitted version.

## Funding

Funding for SB was provided by PhRMA Grant 218218 Pre-Doctoral Fellowship for Pharmacology/Toxicology. NS was partially supported by intramural funding from the Edwards Comprehensive Cancer Center, JCESOM, Marshall University and by the NIGMS under grant number 3P20GM103434-21S2 (WV-INBRE).

## Conflict of Interest

The authors declare that the research was conducted in the absence of any commercial or financial relationships that could be construed as a potential conflict of interest.

## Publisher’s Note

All claims expressed in this article are solely those of the authors and do not necessarily represent those of their affiliated organizations, or those of the publisher, the editors and the reviewers. Any product that may be evaluated in this article, or claim that may be made by its manufacturer, is not guaranteed or endorsed by the publisher.
